# Formulation of non-linear viscoelastic–viscoplastic constitutive equation for polyamide 6 resin

**DOI:** 10.1016/j.heliyon.2021.e06335

**Published:** 2021-02-26

**Authors:** Jun Koyanagi, Kodai Hasegawa, Akio Ohtani, Takenobu Sakai, Kenichi Sakaue

**Affiliations:** a Department of Materials Science and Technology, Tokyo University of Science, Japan; b Department of Materials Science and Technology, Graduate School of Tokyo University of Science, Japan; c Department of Mechanical Engineering, Kyoto Institute of Technology, Japan; d Department of Mechanical Engineering, Saitama University, Japan; e Department of Mechanical Engineering, Shibaura Institute of Technology, Japan

**Keywords:** Viscoelastic, Viscoplastic, Constitutive equation, Polymer, Time-temperature superposition principle

## Abstract

In this study, a non-linear viscoelastic–viscoplastic constitutive equation for polyamide 6 (PA6) is formulated and a new model is suggested for the viscoplastic part of the equation. The suggested model is empirical but can accurately predict the viscoplastic strain. In this study, creep and recovery tests are conducted to evaluate the viscoplastic strain. Using a non-linear dashpot, a viscoplastic strain is formulated and its parameters are identified for PA6. In addition, a stress relaxation test is conducted, and the relationship between the viscoelastic strain and stress is identified when considering the viscoplastic strain. In this study, the time–temperature superposition principle is thoroughly applied to include the effect of elevated temperature on the viscoelastic–viscoplastic behavior. All material constants in the non-linear viscoelastic–viscoplastic constitutive equation including the time–temperature superposition principle for PA6 are presented in this study.

## 1 Introduction

The application fields of polymeric composite materials are widely used not only in the aerospace, energy, and sporting equipment industries, but also in the automobile industry and various types of infrastructure. For these uses, an accurate prediction of the lifetime and residual strength is becoming increasingly important [1, 2, 3]. To assure the long-term durability of the composite materials, their time-dependent behavior should be understood [4, 5, 6, 7, 8]. Because the reinforcing fiber and fiber/matrix interface can be assumed to be time and temperature independent [9, 10, 11, 12], if we know the time-dependent behavior of the matrix, the time-dependent behavior of composite materials can be assumed using micromechanics [13, 14, 15, 16]. The lifetime prediction of a matrix resin is important [17, 18, 19]; therefore, a precise constitutive equation of the matrix is indispensable. In particular, to discuss the time-dependent behavior, we need to consider the time-dependent term in the constitutive equation, that is, both viscoelastic and viscoplastic behaviors. In some studies, the viscoelastic behavior is neglected and thus the numerical implementation into commercial software applied to a finite element analysis, such as ABAQUS, is relatively easy; however, this might be unsuitable for cases discussing long-term durability [13, 20, 21, 22, 23, 24, 25, 26]. Many studies have employed non-linear viscoelastic–viscoplastic constitutive equations to express the time-dependent stress–strain relationships of matrix resins [27, 28, 29, 30, 31, 32, 33, 34, 35, 36]. For example, there have been studies on applying damage as a factor in a non-linear viscoelastic–viscoplastic constitutive equation [37, 38, 39, 40, 41].

Applying micromechanics is an effective way to understand the macro properties of composite materials [13, 14, 15, 42]. Applying the viscoelastic–viscoplastic constitutive equation to a finite element analysis enables us to simulate the micromechanics model; in addition, the macro property can be predicted through a numerical simulation. In a finite element analysis, stress is determined based on strain using the D-matrix. When applying a complicated constitutive equation to a finite element analysis, the D-matrix should be defined in an incremental form with a user-subroutine code. In this implementation, we need to consider that the stress is only derived from viscoelastic strain rather than from viscoplastic strain. In other words, it is extremely important to split the strain increment into viscoelastic and viscoplastic terms. In this sense, a precise formulation of the viscoplastic term is also important; however, there have not been many articles discussing the formulation of the viscoplastic term.

In this study, a thermo-viscoelastic–viscoplastic constitutive equation for a PA6 material is presented, in which we suggest a new model for viscoplastic strain. In this study, the following spring-dashpot model is employed to express the viscoelastic–viscoplastic constitutive equation. Whereas E_1_–E_15_ and η_1_–η_15_ are linear springs and dashpots, respectively, η_vp_ is a non-linear dashpot. Note that this schematic only exhibits a uniaxial term. We also assume that the Poisson's ratio of the material remains unchanged in this study. In this study, we define the strain consisting of the generalized Maxwell model (above part) as a viscoelastic strain, and the strain consisting of a nonlinear dashpot (below part) as a viscoplastic strain. We determine all parameters of this viscoelastic–viscoplastic model in the present study.

**Figure 1:**
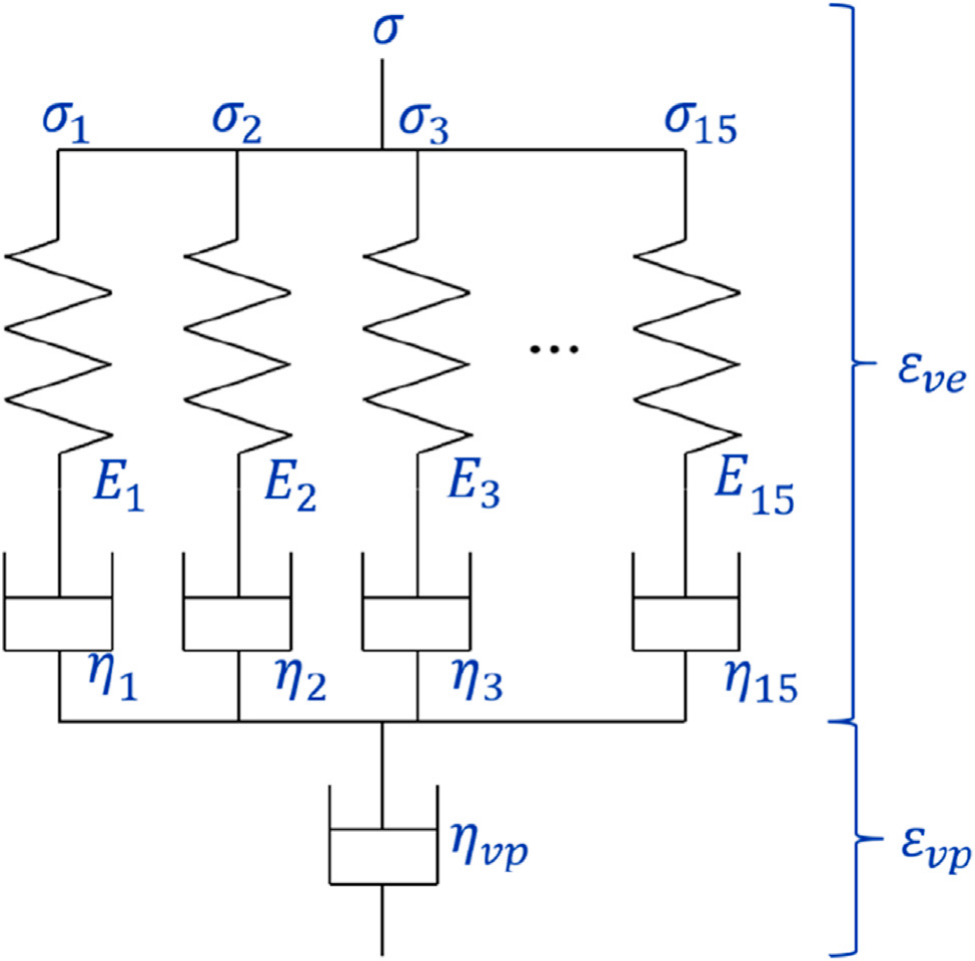
Viscoelastic–viscoplastic model employed in this study.

Note that not power law-type numerical model, which are employed conventionally [2, 9, 10, 11, 28, 37, 43], but the parallel Maxwell model shown in Figure 1 is employed in this study for describing viscoelastic strain, while power law type equation can be suitable to viscoelastic behavior or polymer materials and much more simple than parallel Maxwell model. Because the numerical implementation of power law type constitutive equation into finite element method (FEM) software is a big burden but that of parallel Maxwell model into FEM software is much easier. The number of Maxwell components are equivalent to resolution how the model can predict viscoelastic behavior precisely. Greater number of components is better for the precise prediction but not good for numerical implementation into FEM. In the present study, we employ 15 components as shown in Figure 1.

In this study, we do not consider damage to the materials, and the experiments carried out in this study are assumed to be within the range of intact materials. To separate viscoelastic strain and viscoplastic strain, creep-recovery tests are conducted in this study, and the viscoplastic strain is formulated. Although there are various formulation methods, we consider nonlinear η_vp_, and viscoplastic strain can be obtained from a linear function of time. This is convenient when we construct the D-matrix for a finite element analysis in the future. This non-linear viscoplastic constitutive equation is novel. Subsequently, stress relaxation tests are carried out to formulate the relaxation modulus based on the viscoelastic strain and stress relationship. Note that the viscoelastic strain is determined by subtracting the viscoplastic strain from the total strain. The temperature effect is introduced into a time shift based on the Arrhenius-type time–temperature superposition principle.

## 2 Creep-recovery test

### 2.1 Material

**Figure 2:**
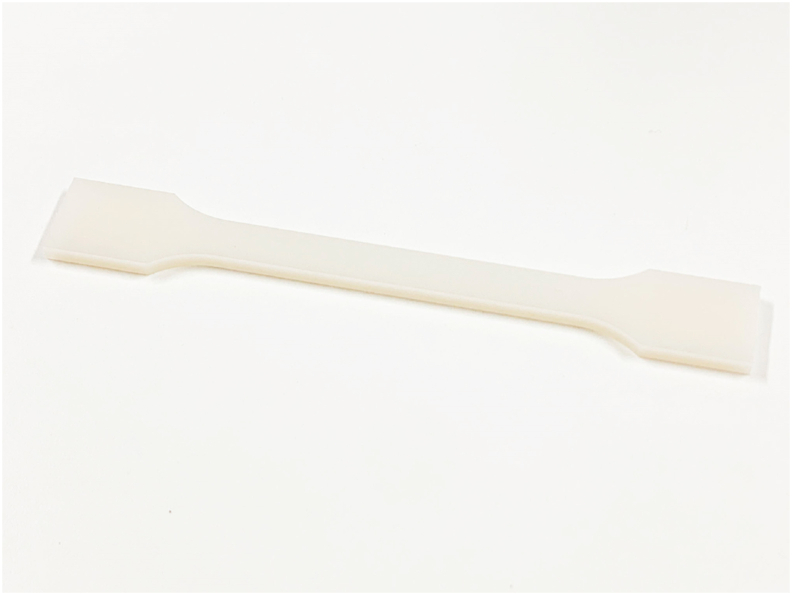
Fabricated PA6 specimen.

PA6 resin (CM1006, Toray Industries, Inc.) is used for injection molding, and a dumbbell-shaped specimen (10-mm width, 4-mm thickness, and 100-mm gauge length) of the JIS K7139-A1 standard is molded (see Figure 2). An injection molding machine (SE75EVA-C110, Sumitomo Heavy Industries, Ltd.) with a clamping force of 750 kN is used. Injection molding conditions are set for a cylinder temperature of 250 °C, an injection speed of 30 mm/min, an injection pressure of 150 MPa, a mold temperature of 90 °C, and a holding pressure of 30 MPa.

### 2.2 Method and results

**Figure 3:**
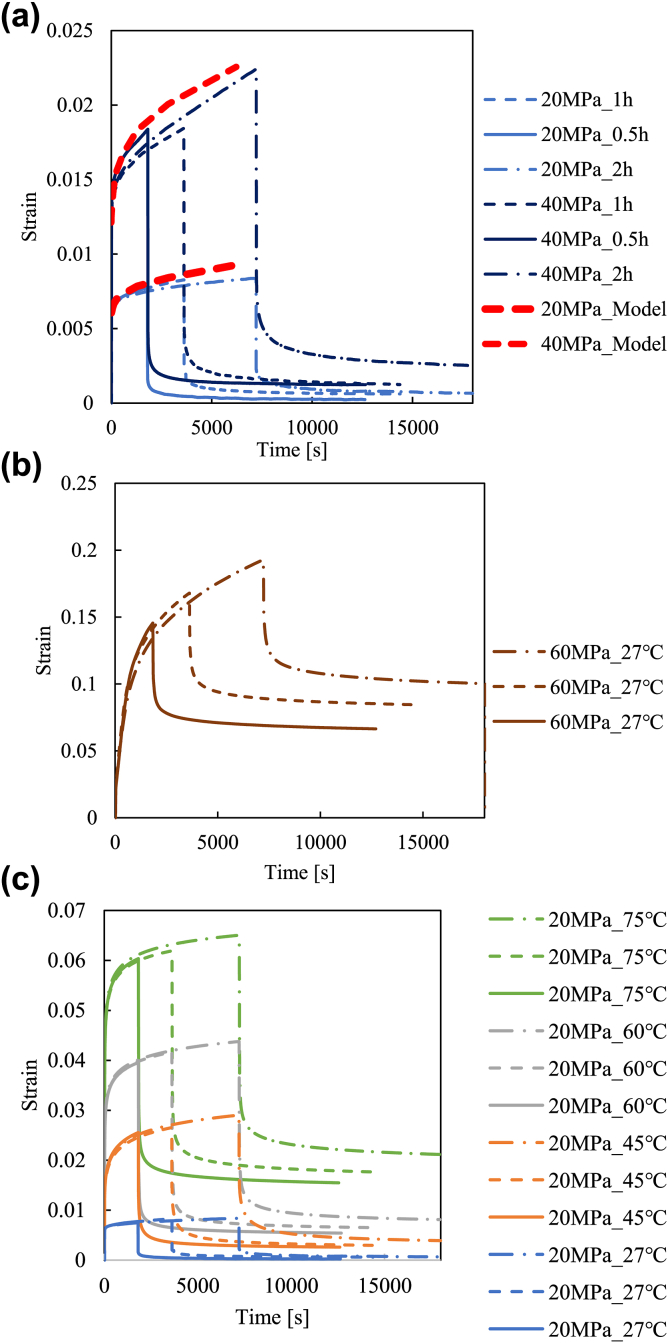
Experimental results for creep-recovery tests for (a) 20 and 40 MPa at ambient temperature (27 °C) with model predictions, (b) 60 MPa at ambient temperature, and (c) 20 MPa at ambient temperature, and at 45 °C, 60 °C, and 75 °C.

Creep-recovery tests are carried out in this study using a universal mechanical testing machine (Shimazu Autograph). The creep stresses are 20, 40, and 60 MPa at ambient temperature (27 °C) and 20 MPa at 45 °C, 60 °C, and 75 °C, respectively. The creep times are 0.5, 1, and 2 h. The experimental results are shown in Figure 3. Note that the creep strain predictions are also depicted in Figure 3(a). They are predicted by nonlinear viscoelastic-plastic model in which the parameters are identified in following section 3 in this paper. The parameter identifications are described later.

For every test, the strain increases with time during creep and decreases with time during relaxation. The strain value is enhanced with the stress value, temperature, and creep time. We can see an irrecoverable strain for each test, which can be regarded as a viscoplastic strain. These are raw data, and these are consistent with each other qualitatively.

### 2.3 Master curve

**Figure 4:**
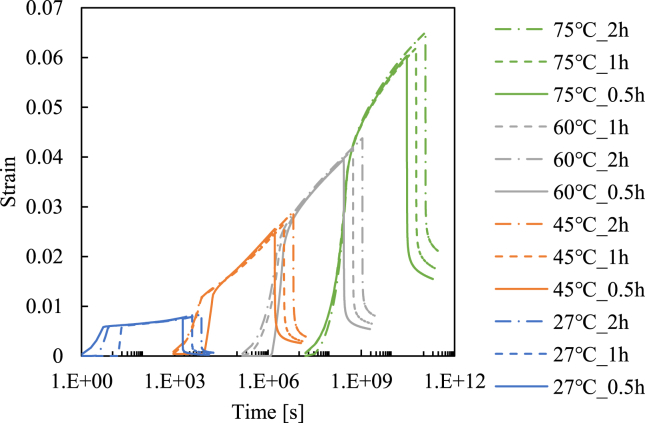
Master curve for 20 MPa creep created by shifting the test results for elevated temperatures.

Based on the Arrhenius-type time-temperature superposition principle, the above test results are shifted along with the log time to construct the creep master curve. The master curve is shown in Figure 4, where the test results at elevated temperature are shifted horizontally based on the following equation.

(1) $$\log\;\alpha_{TR}\left(T\right)=\frac{\Delta H}{2.303R}\left(\frac{1}{T}-\frac{1}{T_{R}}\right)\text{,}$$

where α_*TR*_ is the shift factor, Δ*H* is the activation energy, *R* is the gas constant, *T* is the temperature, and *T*_*R*_ is the reference temperature. We employed *T*_*R*_ = 300 K and R = 8.314 J/K mol. The Δ*H* value for creating a smooth master curve for the creep behavior is determined as 300 kJ/mol, as shown in Figure 4. This value is similar to that presented in [37], and thus we believe this master curve is reasonable.

**Figure 5:**
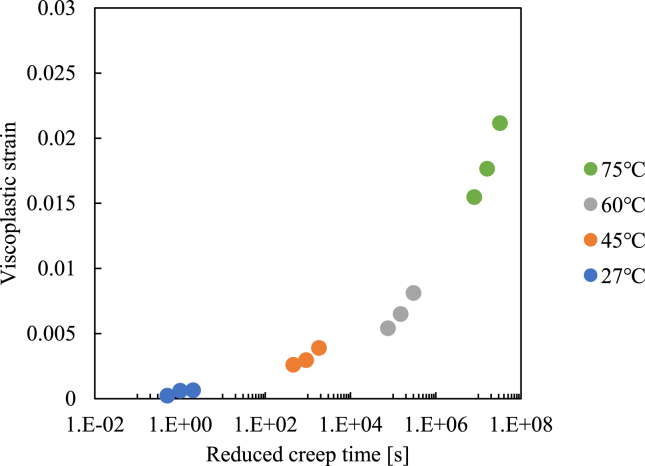
Master plot of viscoplastic strain for 20 MPa creep load.

**Figure 6:**
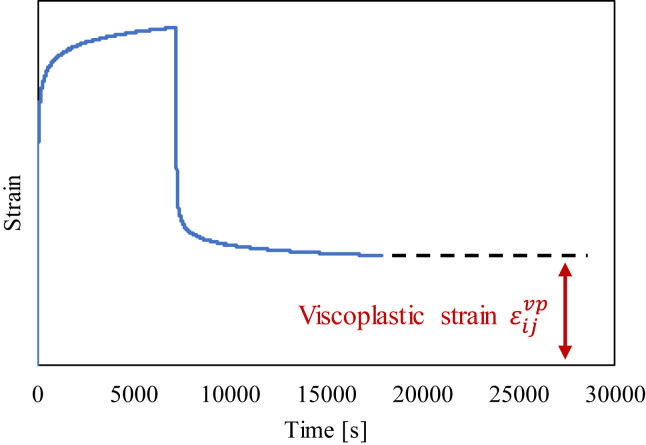
Schematic illustration of method used to determine viscoplastic strain.

Employing the same shift factor, a master curve of plastic strain for 20 MPa creep is obtained, as shown in Figure 5. In this figure, the relationship between the reduced creep time and viscoplastic strain is depicted. We found that the time–temperature superposition principle can also be applied to plastic strain values. Note that the plastic strain for each test can be determined based on the scheme shown in Figure 6. We surmise the extracted asymptotic curve and take saturated values as the plastic strain, as shown in Figure 6.

## 3 Modeling and discussion

### 3.1 Formulation of viscoplastic part and its parameter identification

In this section, we formulate the viscoplastic strain in our viscoelastic–viscoplastic constitutive equation. First, in the present study, the viscoplastic strain is modeled by a nonlinear dashpot, as shown in Figure 1. Therefore, the viscoplastic strain can be expressed through the following simple equation:

(2) $$\varepsilon_{ij}^{\text{vp}}=\int_{0}^{t}{{Η}_{ijkl}^{\text{vp}}}^{-1}\sigma_{kl}dt$$

Here, ***H*** can be written in a matrix form as follows:

(3) $${Η}^{\text{vp}}=\frac{\eta_{\text{vp}}}{\left(1+\nu \right)\left(1-2\nu \right)}\left[\begin{array}{cccccc} \left(1-\nu \right) & \nu & \nu & 0 & 0 & 0\\ \nu & \left(1-\nu \right) & \nu & 0 & 0 & 0\\ \nu & \nu & \left(1-\nu \right) & 0 & 0 & 0\\ 0 & 0 & 0 & \frac{1-2\text{ν}}{2} & 0 & 0\\ 0 & 0 & 0 & 0 & \frac{1-2\text{ν}}{2} & 0\\ 0 & 0 & 0 & 0 & 0 & \frac{1-2\text{ν}}{2} \end{array}\right]$$

Here, *ν* is the Poisson's ratio and *η*_vp_ is determined by the following:

(4) $$\eta_{\text{vp}}=\frac{\eta_{0}\left(1+e^{\beta {\left(\frac{\varepsilon_{\text{eqv}}^{\text{vp}}}{\sigma_{\text{eqv}}}\right)}^{n}}\right)}{\left(1+e^{\alpha \left(\sigma_{\text{eqv}}-\sigma_{0}\right)}\right)}$$

**Figure 7:**
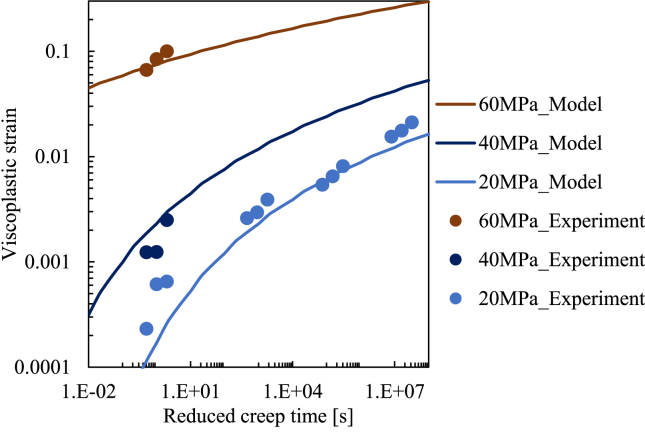
Comparison of experimental viscoplastic strain using model estimation suggested in this study.

Here, subscript eqv is the equivalent value of the current state, *η*_0_ is the initial value of *α*, *β*, and *σ*_0_ and *n* are specific constants determined based on a comparison between the experimental and analytical values. As shown in Eq. (4), *η*_vp_ is expressed as a function of the current equivalent viscoplastic strain and equivalent stress. The value of the denominator increases non-linearly with the current stress when it exceeds a specific stress value of *σ*_0_. The value of the numerator increases non-linearly with the current viscoplastic strain and decreases with the current stress. We found that this empirical form can accurately express the viscoplastic strain in the form of Eq. (2), as shown in Figure 7. As Figure 7 indicates, various experimental results can be reasonably followed by the empirical model suggested in this study. Note that the x-axis is the reduced creep time when considering that the time–temperature superposition principle is applied in Figure 7. The parameter values used for the model in Figure 7 are listed in Table 1. Thus, we conducted a formulation and parameter identification for the viscoplastic part of our viscoelastic–viscoplastic constitutive equation for PA6.

**Table 1:** Parameter values for viscoplastic part.

*η*_0_	58000000/MPa
*α*	0.89
*β*	385
*σ*0	35MPa
*n*	0.43

### 3.2 Formulation of viscoelastic part and its parameter identification

**Figure 8:**
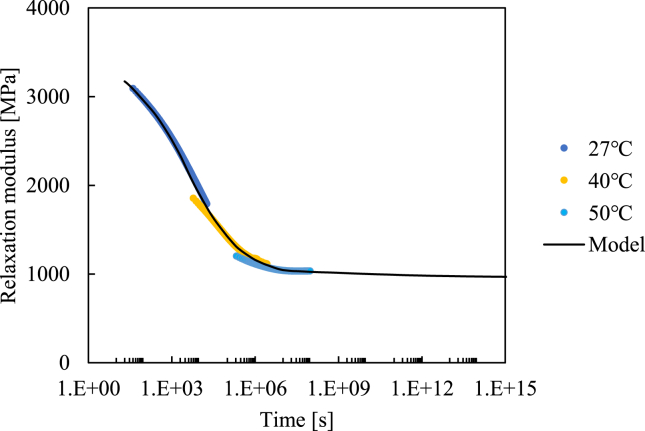
Relationship between relaxation modulus and reduced time.

In this section, we formulate the viscoelastic part of our constitutive equation. Stress relaxation tests were carried out in this study. The same specimen as in the previous section was used for the stress relaxation tests. The tests were carried out at ambient temperature (27 °C) and at 40 °C and 50 °C. To evaluate a linear viscoelastic constitutive equation, the tests were conducted within a relatively low stress of approximately 10 MPa. The test results are shown in Figure 8, in which the x-axis exhibits a reduced time based on the same time–temperature superposition principle as in the previous section, that is, the same activation energy value is used. The value of the relaxation modulus is defined as follows:

The tests were carried out under a fixed displacement. However, in reality, the specimen strain changed slightly over time, and we measured it using a strain gauge. Based on Eq. (2), the viscoplastic strain as a function of stress and time can be obtained such that the viscoelastic strain can be obtained by subtracting the viscoplastic strain from the measured strain. The relaxation modulus is determined by dividing the measured stress by the viscoelastic strain at that specific time. Note that, although it is assumed in this study that the relaxation modulus can be evaluated using this test, the strain is consequently not constant, and the result obtained might not completely relax the modulus. We can also see the master curve for the relaxation modulus, which means that the activation energy taken in this study is reasonable.

The relaxation modulus predicted in this study is also shown in Figure 8. We assume the spring-dashpot model, as shown in Figure 1, and the constants employed for this fitting listed in Table 2. In order to cover significantly wide range of time, the values of dashpot *η*s are increased approximately exponentially. After that the values of spring *E*s are determined by comparison with experimental results. Thus, we have formulated and identified every parameter for the non-linear viscoelastic–viscoplastic constitutive equation for PA6 in a form that can be readily implemented in a general finite element analysis, for which a new equation is suggested for the viscoplastic part. In a following study, we will utilize this constitutive equation to express the time- and temperature-dependent constitutive equation for PA6.

**Table 2:** List of constants employed in this study.

Spring	MPa	Dashpot	/MPa
E_1_	255	η_1_	10000
E_2_	305	η_2_	90000
E_3_	515	η_3_	1100000
E_4_	470	η_4_	5200000
E_5_	380	η_5_	27000000
E_6_	203	η_6_	88000000
E_7_	132	η_7_	4.7E+08
E_8_	17	η_8_	1.1E+09
E_9_	12	η_9_	1.3E+10
E_10_	11	η_10_	9E+10
E_11_	11	η_11_	1.1E+12
E_12_	8	η_12_	8E+12
E_13_	6	η_13_	9E+13
E_14_	5	η_14_	1E+15
E_15_	970	η_15_	6E+31

Consequently, all the parameters consisting of non-linear viscoelastic-viscoplastic constitutive equation are determined. Now we can predict arbitrary stress-strain-time relationship using this model. We predict the strain-time relationship under creep loading and the predictions are shown in Figure 3(a). As shown in this figure, strain-time relationships are reasonably predicted by the model suggested in the present study, for the range in which the materials are intact, i.e. at low stress level. Note that this model can be also used for cyclic loading condition [35, 36], but this model assumes the intact material, so we have to be careful when material damage occurs during cyclic loading. For such cases, we need to introduce damage parameter in this model, which is our future work.

## 4 Conclusion

In the present study, a non-linear viscoelastic–viscoplastic constitutive equation for polyamide 6 (PA6) is formulated in which the new empirical model is suggested for the viscoplastic part in the equation. The material constants of the empirical model viscoplastic behavior is identified through creep and recovery tests. The effect of elevated temperature on the time progress is considered through the Arrhenius-type time–temperature superposition principle. Based on the results, the relationship between the viscoelastic strain and stress is identified by the displacement relaxation test. The identical time–temperature superposition principle can be applied to the creep master curve, viscoplastic strain, and viscoelastic strain. Including the activation energy, all material constants for the suggested non-linear viscoelastic–viscoplastic constitutive equation are presented in this study.

## Declarations

### Author contribution statement

Jun Koyanagi: Conceived and designed the experiments; Analyzed and interpreted the data; Wrote the paper.

Kodai Hasegawa: Performed the experiments; Analyzed and interpreted the data.

Akio Ohtani: Conceived and designed the experiments; Performed the experiments.

Takenobu Sakai & Kenichi Sakaue: Analyzed and interpreted the data; Contributed reagents, materials, analysis tools or data.

### Funding statement

This work was supported by JST-Mirai Program Grant Number JP19215408, Japan.

### Data availability statement

Data included in article/supplementary material/referenced in article.

### Declaration of interests statement

The authors declare no conflict of interest.

### Additional information

No additional information is available for this paper.
